# Generative artificial intelligence in the publishing industry: adoption, use, intellectual property, and other challenges

**DOI:** 10.3389/frma.2026.1759242

**Published:** 2026-03-02

**Authors:** Marco Giraldo-Barreto

**Affiliations:** Publishing Department, Universidad de Bogotá Jorge Tadeo Lozano, Bogotá, Colombia

**Keywords:** academic publishing, authorship, creative industries, generative artificial intelligence, intellectual property, publishing industry

## Abstract

Taking as a starting point how generative artificial intelligence (GenAI) works, this text explores the level of adoption of such technology in the publishing sector (in particular for Latin America), shows examples of legislation challenges faced by states and the publishing industry in terms of intellectual property, and the implications of GenAI misuse in the academic publishing context. Finally, it proposes a course of action for a responsible adoption for the publishing chain of value.

## 1 Introduction

In recent years, since the emergence of generative artificial intelligence (GenAI) on the market, expectations regarding its capabilities and concerns about its impact have become recurrent themes in both the news and the academic literature. Although the topic has been a matter of study in Computer Science for more than 70 years, the current boom is totally unprecedented; given the development of technology, GenAI has achieved today what could not have been possible a few years ago.

One of the main points of discussion currently concerns the use and abuse of AI in the creative industries, particularly in the publishing sector, as well as the intellectual property of the works created using AI. Regarding the first point, in June 2025, the Regional Center for the Promotion of Books in Latin America and the Caribbean, Cerlalc, published the report *Navegando lo incierto. Usos y percepciones de la IA generativa en el sector editorial iberoamericano* (*Navigating uncertainty: uses and perceptions of GenAI in the Ibero American Publishing Industries*) (Dujovne et al., 2025). The document summarizes the results of a survey conducted from October to December 2024 among people working in the publishing industry. According to the report, 50% of the people surveyed have used GenAI for their everyday work, and 54% of the people are interested in acquiring knowledge and training in the use of these tools. Major concerns about intellectual property and creative work are expressed by illustrators and translators, while people in distribution, developmental editing, or proofreading report a reduction in their workloads or a more frequent use of GenAI in their activities. There is a particularity, though, among translators and illustrators: most of them are not willing to use AI, even though they are not familiar with it. Big questions arise: What makes a professional in the industry so reluctant to use GenAIs? What are their principal concerns? Are those concerns factual or just intuitions?

The top-of-mind answers to those questions revolve around intellectual property. The media has reported how the use of copyrighted work for training such systems is unclear, and how companies had access to such works. This article will explore how GenAIs have had an impact on the publishing industry, particularly in book publishing and academic publishing; explore some of the legal challenges faced by governments and creators; and propose a course of action to make responsible use of GenAIs in the upcoming years.

## 2 Understanding GenAI

(Champagne and Shear 2024, p. 1) state that AI models “are not built through knowledge, but by statistically evaluating billions of samples of text to simply answer: given a series of words, what word is most likely to come next?” Furthermore, according to (Dien 2023, p. 1), “the algorithm is a method for condensing and summarizing vast quantities of text written by humans. Such algorithms typically are given a large training set, in this case downloaded from the Internet, which detect repeating patterns of words. This distillation is the foundation upon which the algorithm bases its output. The GPT text model is then accessed by ChatGPT, which provides a natural language interface.”

The ability of a GenAI to produce text does not stem from intelligence itself, but from the prediction of patterns. According to (Chomsky 2009, p. 76), “human language (…) is free from the control of independently identifiable external stimuli or internal states and is not restricted to any practical communicative function (…). It is thus free to serve as an instrument of free thought and self-expression.” Most importantly, Chomsky states, “Language provides finite means but infinite possibilities of expression constrained only by rules of concept formation and sentence formation, these being in part particular and idiosyncratic but in part universal” (p. 76). In contrast, “Symbolic AI systems require human experts to encode their knowledge in a way the computer can understand. This places significant constraints on their degree of autonomy. While they can perform tasks automatically, they can only do so in the ways they are instructed, and they can only be improved by direct human intervention” (Boucher, 2020, p. 3). GenAIs process large amounts of data and, as a statistical result, a text is generated when some input is given. What is surprising—and perhaps the most effective marketing strategy—is that the result is coded in a way that is comprehensible for a person looking at the screen. This communicative familiarity, developed using a natural language structure, has convinced many people that GenAIs such as ChatGPT, Deepseek, or Claude are infallible.

Such a strategy, plus the global context in which innovations in technology become widely accessible to people around the world with an Internet connection, has helped people elude the fact that these kinds of systems have several limitations and biases. During the growth and peak of usage in 2023, ChatGPT warned its users about its limitations, including limited information before 2021, harmful instructions or biased content, and occasional incorrect information. Ray (2023) characterized more than 20 limitations and biases of the tool, including cultural, linguistic, gender, racial, content recommendation, ideological, clickbait, and commercial biases, and limitations such as misleading information, overuse of phrases, lack of contextual awareness, overgeneralization, and inconsistency in quality, among others, many of which are still being reinforced in those systems. Terms of use of GenAIs such as ChatGPT warn users on the issue: “Our services may provide incomplete, incorrect, or offensive Output,” “Output may not always be accurate. Your should not rely on Output from our services as a sole source of truth or factual information, or as a substitute for professional advice” (OpenAI, 2024).

## 3 Legislation challenges

Regarding GenAI, legal issues are usually the principal concern among creators, publishers, creative industries, and entrepreneurs. According to Dujovne et al. (2025), the profiles most concerned with copyright infringement and GenAI are illustrators (74%), translators (60%), designers (47%), and writers (45%). “Data reflects that such concern does not affect only writing, translation, and illustration professionals, but also people who participate in book production and distribution. The lack of transparency and information of big corporations that develop IA models toward the resources used for training such systems seem to strike a chord and create uncertainty^1^” (Dujovne et al., 2025, p. 56).

(Con Díaz 2025) provides some context for the legal evolution of intellectual property and copyright concerns by explaining the Google Books project at its early stages. Initially conceived as a way to digitize books from different universities in the US, “On September 20, 2005, the Authors Guild—the largest trade group for authors in the country—files a class action lawsuit against Google to stop the library program. The complaint alleged that Google was committing infringement because it was violating (among other things) authors' exclusive right to reproduce, distribute, and display their work” (p. 194), which is no different from the current legal lawsuits against OpenAI and Anthropic, owners of ChatGPT and Claude. In fact, Milliot (2025) reported that “Following a September 25 hearing, U.S. District Judge William Alsup gave preliminary approval to 1.5 billion settlement in the class action lawsuit in which authors charged AI giant Anthropic with copyright infringement for using pirated books to train its large language models.”

The use and abuse of copyrighted material is at the core of the controversy, but it is not the only concern. In 2023, Jane Friedman, former president and CEO of HarperCollins and contributor to The New York Times, The Atlantic, CNN, and NPR, among many other important companies, published on her blog that she was a victim of fraud. AI-generated books were being sold on Amazon under her name, and for a while, she was incapable of taking them down. As Amazon said, she did not have any copyright over such books, as stated by herself; consequently, the supposed copyright ownership could not be asserted (Friedman, 2023). What happened to Friedman is dangerous, especially at times when technologies run faster than legal frameworks and debates on AI authorship remain unclear, not only in public discussions but also in the law.

In terms of how legislation has evolved in recent times, the first legislation concerning the use of AI comes from the European Union. Regulation (Eu) 2024/1689 of the European Parliament and of the Council classifies AI systems into four risk categories (minimal, specific transparency, high, and unacceptable). It also presents specific rules for models such as GPT or Claude and “provides developers and deployers with clear requirements and obligations regarding specific uses of AI while reducing administrative and financial burdens for businesses” (European Commission, 2024). In contrast, the decisions to be made regarding AI regulation in the US are not nationwide but federal, which poses difficulties for both users and companies in terms of the reach, impact, and liability.

One example of how the lack of understanding of how GenAI works and the gold rush related to that technology, as well as a lack of clear legislation, can be taken from the attempt of the Colombian Congress to include in a bill reform project to recognize AI as an author: “Article 2. The following definitions will be implemented in this bill (…). Author. Person or Intelligence producer of a text, whether fictional or informative” (Congreso de Colombia, 2024). Although the bill reform was in the end archived, it almost caused irreparable damage to authors and to the book industry in the country. Congressmen did not have in mind that such a definition contradicted the definition of author included in Decision 351 of the Andean Community, to which Colombia subscribed: “Author: physical person that performs the intellectual creation.” Additionally, under Colombian law, in order for copyright to apply, all works must be original (i.e., not a copy of another work), cannot be the result of randomness but of the creative effort of a person, and must have a support to be reproduced. In such a sense, no GenAI can be legally considered an author, nor can it have any legal status of creator, and, in consequence, no copyright protection can be guaranteed for GenAI creations, unless GenAI is used as a mere tool and the intellectual work of a person is noticeable in the result. Although the conclusion is evident, the current debates concerning ownership still shape the public discussion, and, in the end, the market moves faster than regulations.

## 4 Hallucinations and academia

Although the following cases are not related to the book industry, they reflect the impact of GenAI in academic and scientific publishing contexts. Manohar and Prasad (2023) reported that, for a study on systemic lupus erythematosus, they used ChatGPT to evaluate the usefulness of GenAI in academic publishing. According to them, although they noticed that the text was readable and coherent at first sight, “at closer inspection, text that appeared fluent and informative did not really provide scientific data. It was legible, sure, but far from the requirements of academic writing. The citations were duplicated, and most of them did not link to real work” (p. 6–7).

Such results might be described as AIs' hallucinations, which are defined as erroneous output (Dien, 2023). According to Champagne and Shear (2024, p. 1), “Over time, new LLm being trained on new data and information drawn from the Internet run the risk of being trained on LLM-generated material in a vicious feedback cycle of hallucinatory non-sense (Shumailov et al., 2023) (…) When an LLM is trained for what word should come next in this sequence, if it is training from text that was already generated by another imperfect LLM generating imperfect sequences, these studies suggest that it leads inexorably into absurdity.”

In the medical context shown above, according to Manohar and Prasad (2023), a concern is raised: “publishing such convincing text with non-existing citations can lure persons into a world of misinformation that can alter their perceptions of healthcare practices.” (p. 7). With the recent popularization of ChatGPT and other GenAIs as a free tool and even included in certain devices, concerns have been proven to turn into real dangers.

In March 2024, an article by Zhang et al. was retracted from an Elsevier journal. The primary reason, as stated in the retraction notice, was that “there are concerns that the authors appear to have used a Generative AI source in the writing process of the paper without disclosure, which is a breach of journal policy.” Evident concerns were already present, as the very first sentence of the paragraph indicated the use of GenAI: “Certainly, here is a possible introduction for your topic.” The question that remains is how neither the authors, proofreaders, editors, peers, nor anyone involved in the publishing process noticed that the text was generated by GenAI. Furthermore, this incident shows that, despite journals having policies regarding the use of GenAI, no system is infallible, and breaches can occur.

Scientists, academic authors, editors, peers, and publishers who work with science have a responsibility to their audiences. Scientists from all around the world trust these journals because they are supposed to follow high standards in their processes. Keeping in mind that GenAI tools can be biased and limited, a detail like that not only raises questions regarding the writing process but also the research process itself. How much information was given by GenAI? Was that information real or hallucinatory? Why was the use of GenAI not mentioned? Was GenAI used to improve the style and writing, or was it used with the rush given by the motto in academic publishing, *publish or perish*? What was the role of peers and editors when revising, and why did they let it pass? Can audiences trust authors, peers, or the journal itself? The article was retracted, but the damage was done.

## 5 Discussion

At this point, it is undeniable how fast the adoption of these new technologies has occurred. Two years ago, questions such as how good these technologies would be, how fast the legal framework would catch up, and what the market would dictate made a lot of sense; in recent times, and as described above, these technologies are impossible to be considered totally reliable and infallible, not only for the generation of content itself but also for the use some people have given to GenAI to deceive. The frameworks are still under construction and debate, and some of them have put aside basic principles, such as human rights and inherent human qualities, in the legal discussion in order to open the road to the interests of the industry.

However, regarding the publishing industry, and according to Dujovne et al. (2025, p. 55), “the writing, translation and illustration occupations share creativity, imagination, technical capability, and human intentionality as a central axis of their work. In such sense, the question about if AI can empower or enrich creative processes or if, under the demands of the market, it will end up gradually replacing human creativity with automatized processes generated by models specifically trained to imitate and replicate the creative human condition. Then, the risk of an standardized cultural production appears, eliminating the particularity and complexity of human creation”^1^. In such a sense, it is worth remembering that intellectual property is a universal human right: “2. Everyone has the right to the protection of the moral and material interests resulting from any scientific, literary or artistic production of which [they are] the author” (Universal Declaration of Human Rights, Article 27) (United Nations, n.d.). If authors and creators around the globe have concerns regarding GenAI, it is because a human right is at stake. As seen before, GenAI companies have been used for their alleged infringement of copyrighted work; thus, that is not only a discussion of economic profit but also of how human rights have become vulnerable in regard to these new technologies.

What might be, then, the most effective course of action for the appropriation of GenAI in the industry while being respectful of intellectual property and of the publishing actors' work?

1. Education. The publishing industry still has a road to go. Illustrators, translators, and writers still show concerns regarding AI biases, content standardization, quality reduction, job loss, and the level of impact on the diversity of actors in the industry. To overcome such fears, the industry must educate those actors and every other professional in need of training, which might come in the form of teaching local and international regulations, critical thinking regarding ethical use of AI, prompting, implementation of AI in workflows, and even the environmental impact of AI for companies or users that measure their carbon impact. Working hand-in-hand with universities and technical education institutions, and promoting state education in areas concerning intellectual property, GenAI, and GenAI management, might be the starting point for professionals to overcome their fear of AI and benefit from it.
2. Self-regulation. Common laws are slow, hard, and costly to implement. In this context, clear guidelines for writers, illustrators, professional readers, and evaluators could help establish a common ground of good practices, which in turn can be shared institutionally. Book chambers of commerce, book fairs, and the media can help not only to draft manuals or proceedings for GenAI good practices but also to centralize information, procedures, and good results in order to be replicated in such scenarios. A good starting point for good practices might come from the general guidelines of the EU AI Act and local studies of adoption and impact, such as Cerlalc's, but applied to a wider number of professionals in local contexts.
3. Follow-up. As the Cerlalc report demonstrated, surveys of the sector are important, but just one will not be enough to understand the effects of GenAI in the industry. The impacts of AI are still to be observed, and they will not be uniform around the globe. Subsequent research regarding education, the evolution of the technology, the evolution of the legislation, and economic impact is encouraged and should be performed by local governments in order to identify opportunities for economic development for all of the actors in the publishing chain of value.
4. Cooperation. To educate, implement nationwide laws, and enhance research that leads to economic development for the actors in the publishing industry, cooperation among instances is essential. Private corporations, the public sector, universities, and individual actors can expand the scope of policies and actions while reducing the time, effort, and technological gaps currently affecting the industry.

## Data Availability

The original contributions presented in the study are included in the article/supplementary material, further inquiries can be directed to the corresponding author.

## Footnotes

1 Fragment translated by the author. Original text available in Spanish.
